# The effects of free condom distribution on HIV and other sexually transmitted infections in men who have sex with men

**DOI:** 10.1186/s12879-019-3839-0

**Published:** 2019-03-04

**Authors:** Reinier J. M. Bom, Kalja van der Linden, Amy Matser, Nicolas Poulin, Maarten F. Schim van der Loeff, Bouko H. W. Bakker, Theodoor F. van Boven

**Affiliations:** 1 Condomerie, Amsterdam, the Netherlands; 2 Department of Research, Cluster of Infectious Diseases, Public Health Service of Amsterdam (GGD Amsterdam), Amsterdam, the Netherlands; 3 Julius Center for Health Sciences & Primary Care, University Medical Center Utrecht (UMCU), Utrecht University (UU), Utrecht, the Netherlands; 4 Centre de Statistique de Strasbourg (CeStatS), Institut de Recherche Mathématique Avancée (IRMA, UMR 7501), Université de Strasbourg & Centre National de la Recherche Scientifique (CNRS), Strasbourg, France; 5 Center for Infection and Immunology Amsterdam (CINIMA), Academic Medical Center (AMC), University of Amsterdam (UvA), Amsterdam, the Netherlands; 6 Rutgers, Utrecht, the Netherlands

**Keywords:** Behavioural interventions, Condoms, Cost effectiveness studies, Men who have sex with men, Models/projections, Prevention of sexual transmission, HIV, STI, Hepatitis C, Chlamydia, Gonorrhoea, Syphilis

## Abstract

**Background:**

HIV and other sexually transmitted infections remain a burden on men who have sex with men in the era of effective combination antiretroviral therapy. New prevention efforts are therefore needed. One of these approaches is the current country-wide free condom distribution at gay bars with darkrooms and gay saunas in the Netherlands. This study assessed the effects of free condom distribution on incidence and burden of disease of HIV and other sexually transmitted infections.

**Methods:**

A static model was constructed to calculate the impact of free condom distribution on HIV, hepatitis C, chlamydia, gonorrhoea, and syphilis among men who have sex with men visiting these venues. Outcomes included new infections averted and disability-adjusted life years averted. Scenario studies were performed to predict the effects of a further increase of condom use, condom effectiveness and coverage. Lastly, cost-effectiveness and sensitivity analyses were performed.

**Results:**

Our model showed that condom use at public sex venues increased after the intervention. Annual incidence risk decreased, ranging from 5.73% for gonorrhoea to 7.62% for HIV. The annual number of new infections averted was largest for chlamydia and gonorrhoea (261 and 394 infections, respectively), but 42 new HIV infections were averted as well. In scenarios where condom use and condom effectiveness were further increased, the number of infections reduced more extensively. Over 99% of the decrease in burden of disease was due to HIV. The intervention was cost-effective and cost-saving (for every €1 spent on condom distribution, €5.51 was saved) and remained this in all sensitivity analyses.

**Conclusions:**

Free condoms at public sex venues could reduce the transmission of HIV and other sexually transmitted infections. Condom distribution is an affordable and easily implemented intervention that could reduce the burden of disease in men who have sex with men substantially.

**Electronic supplementary material:**

The online version of this article (10.1186/s12879-019-3839-0) contains supplementary material, which is available to authorized users.

## Background

Promoting condom use has been part of HIV prevention strategies since the beginning of the epidemic among men who have sex with men (MSM) [1]. As the efficacy (the effects under ideal conditions) and the effectiveness (the effects under real-life conditions) of condoms in reducing HIV transmission became apparent during the 1980s, their use was actively promoted, which lead to a steady increase in condom uptake [2]. By the end of the decade, brands of condoms especially designed for anal intercourse were marketed to MSM. As the use of condoms and other safe sex practices became common, the incidence of HIV dropped sharply [3]. This lower incidence was maintained throughout the 1990s, until the introduction of effective combination antiretroviral therapy (cART) in 1996. New antiretroviral therapies greatly improved the quality of life and life expectancy of those living with HIV, but also led to decreased sexual risk perception among MSM [4–8]. As a consequence, sexual risk behaviour, such as unprotected anal intercourse (UAI), has increased among MSM, and prevalences of various sexually transmitted infections (STIs) have risen [9, 10]; also the incidence of HIV increased [3].

Gay bars with darkrooms and gay saunas (collectively known as public sex venues or PSVs) are known to attract both seropositive and seronegative MSM. UAI with anonymous partners is not uncommon at PSVs [11]. Therefore, these locations are major risk locations for serodiscordant mixing and possible transmission of HIV and other STIs. Availability and promotion of free condoms could increase condom uptake, and therefore decrease transmission. In response to decreasing use of condoms and increasing sexual risk behaviour, a collaboration of various PSVs in Amsterdam, sexual health promotion institute Schorer, and public health service GGD Amsterdam started to promote safe sex in PSVs. Free condom distribution is an integral part. At first, condoms and lubricants were paid for by PSVs and offered for free to their patrons, which showed to be a financial burden for many PSVs, and therefore has been an obstacle for upscaling and structural implementation. These initial problems were resolved as condom distribution was expanded into the *CLub GUN* (*Condoms & Lubricant Gay United Netherlands*) project by AIDS Fonds (Dutch AIDS fund) and Condomerie (retail, wholesale and knowledge centre of condoms and lubricants) through the offering of subsidised condoms and lubricants [12]. Based on the Amsterdam experience, the intervention was implemented nationwide and is currently being supported by sexual health promotion institute SOA AIDS Nederland and local public health services. At the start of 2014, 32 PSVs in 11 different cities throughout the Netherlands were participating in *CLub GUN* and were offering free condoms and lubricants to their patrons. A pilot study was conducted on the feasibility of free condom distribution at PSVs in Amsterdam and Rotterdam between 2006 and 2008 [11]. Availability of free condoms was greatly appreciated and most patrons reported a positive influence on condom use. About half of the patrons started to make use of free condoms within the first weeks and 14% of them reported they would not have used a condom, if condoms had not been freely available at PSVs.

Although free condoms and lubricants are now available at most PSVs throughout the Netherlands and similar initiatives are seen internationally [13, 14], the effects of these kinds of interventions on the transmission of HIV and other STIs were never investigated. Therefore it is unknown to what extent free condom distribution can reduce the burden of these infections in MSM. To assess this, we developed a static model to calculate the incidence of HIV before and after free condom distribution at PSVs. In addition, we performed a cost-utility analysis to estimate the number of new infections averted (NIA) and the reduction in burden of disease among MSM by calculating disability-adjusted life years (DALYs) averted. Next to HIV, annual incidence risk, NIA and DALY were calculated for hepatitis C, chlamydia, gonorrhoea and syphilis. Scenario studies were performed to assess the effects of increasing condom use, condom effectiveness and coverage. Lastly, cost-effectiveness and sensitivity analyses were performed.

## Methods

### Data collection and parameters

To investigate possible effects of free condom distribution at PSVs, we used data from three main sources. The first data collection we used came from the study “*Gezonde keuzes makkelijk maken (Making healthy choices easy)*”, the pilot study on free condom and lubricant distribution, conducted by Schorer and GGD Amsterdam [11]. In this study, 375 patrons from PSVs in Amsterdam and Rotterdam were interviewed before the intervention, and 1010 men were interviewed afterwards, between December 2006 and May 2008. The second data source we used was the “*MSM network study”* conducted among 2492 MSM at the STI outpatient clinic of GGD Amsterdam between July 2008 and August 2009 [15, 16]. The third one was the nationally representative study *“LHBT Survey 2013”*, conducted among 883 MSM in June and July 2013 by Rutgers [17]. As the current study made use of previously published datasets, no ethical approval was required. Statements on ethical approval were provided in the subsequent publications, if applicable. All key parameters are presented in Table 1, and a detailed description of extraction of parameters can be found in Additional file 1: Data 1.

**Table 1:** Key parameters used in the model

Parameter	Point estimate	Uncertainty	Source
Condom use in PSVs after intervention	87.8%^a^	83.8, 91.0%	[11]
Percentage of MSM that used a condom that were influenced through the availability of free condoms at PSVs	49.4%^a^	43.9, 54.8%	[11]
Percentage of MSM that used a free condom from the condom distribution, and that reported they would not have used a condom if condoms were not freely available at PSVs	14.2%	9.4, 20.3%	[11]
Percentage of relationships with casual partners that involved anal intercourse formed at PSVs among men frequenting PSVs	59.6%^a^	56.5, 62.7%	[16]
Number of active MSM in the Netherlands	300,000	estimate	[33]
Percentage of active MSM, who met a casual partner at a PSV in the last year	17.1%^a^	15.5, 18.9%	[17]
Number of condoms distributed in 2013	371,952	exact	[34]
Condom wastage	15%	estimate	
Mean annual number of casual partners among men frequenting PSVs	21.8^a^	1–200	[17]
Mean number of sexual acts per casual partner	2.20^a^	1–50	[16]
Percentage insertive anal intercourse per sexual act	63.3%^a^	59.2, 67.2%	[16]
Market share of *CLub GUN* condoms at the *CLub GUN* locations	79.3%	73.8, 84.0%	
Condom effectiveness	70%	estimate	[35, 36]
Prevalence among casual partners met at a PSV			
HIV	36.2%^a^	29.9, 42.8%	[16]
Hepatitis C	0.46%^a^	0.05, 2.11%	[16]
Chlamydia	10.0%^a^	6.6, 14.5%	[16]
Gonorrhoea	5.9%^a^	3.4, 9.7%	[16]
Syphilis	1.8%^a^	0.6, 4.3%	[16]
Prevalence among casual partners met in general			
HIV	22.8%^a^	19.9, 26.0%	[16]
Hepatitis C	0.40%^a^	0.11, 1.08%	[16]
Chlamydia	9.6%^a^	7.6, 11.8%	[16]
Gonorrhoea	6.1%^a^	4.5, 8.0%	[16]
Syphilis	2.0%^a^	1.2, 3.2%	[16]
Per-act infectivity			
HIV	1.025%	estimate	[37]
Hepatitis C	0.5%^a^	estimate	[38]
Chlamydia	17%	estimate	[39]
Gonorrhoea	50%	estimate	[40]
Syphilis	30%	estimate	[41]

*PSV* public sex venues, *MSM* men who have sex with men

Uncertainty is given as 95%CI (beta distribution), except when given otherwise

^a^Additional analyses were done to come to the parameter values provided

### Incidence

Annual incidence risk before and after the introduction of free condoms at PSVs were calculated for HIV, hepatitis C, chlamydia, gonorrhoea, and syphilis. A detailed description of these calculations can be found in Additional file 2: Data 2. To estimate number of incident cases, we multiplied number of susceptible men by annual incidence risk. For HIV, only HIV-negative men were assumed susceptible for infection. For hepatitis C only HIV-positive MSM were assumed susceptible [18]. All men were assumed susceptible for chlamydia, gonorrhoea and syphilis. NIAs were calculated by subtracting the number of incident cases after the intervention from those before.

### Burden of disease

DALYs of the various STIs were calculated using uniform age weights and without discounting [19, 20]. Generalised courses of infection of HIV, hepatitis C, chlamydia, gonorrhoea, and syphilis in Dutch MSM in presence of treatment were constructed and included in the models, using the data of the *Burden of Communicable Diseases in Europe* (*BCoDE*) project [21]. These included the different stages of infection a patient passes through, the experienced disability within the various stages and their durations. Parameter estimates of these generalised courses of infection within this risk group were collected from literature [21]. A detailed description of these generalised courses of infection and parameter estimates can be found in Additional file 3: Data 3.

### Condom use, condom effectiveness, and coverage

Scenario studies were performed to examine the effects of increasing condom use and effectiveness. We constructed a scenario in which all participants increased their condom use at PSVs to 100%, while all other factors remained the same. Similarly, we constructed a scenario where the condom effectiveness at PSVs increased to 100%, while all other factors remained the same. Next, we constructed a scenario where both condom use and condom effectiveness at PSVs were 100%.

In addition, we calculated how much of the susceptible MSM in the Netherlands the condom distribution program currently covers, and what the effects would be if the intervention would covered 100% of the Netherlands. Lastly, we calculated how much condoms were needed when both condom use and coverage are 100%, which is the maximum number of condoms possibly needed.

### Cost-effectiveness analyses

To assess the cost-effectiveness of the intervention, we calculated the total and maximal costs of the intervention. These included purchase, storage, distribution of condoms and lubricants used, plus additional costs such as salary and promotion costs. From these data we calculated costs per DALY, by dividing the total costs by the total number of DALYs averted. World Health Organization (WHO) criteria were used to assess cost-effectiveness [22]. To assess whether the intervention is cost-saving, we calculated the potential annual costs per averted HIV infection. Costs of bacterial infections and hepatitis C were excluded. No data were found on which proportion of bacterial infections will remain untreated and therefore the total costs cannot be calculated. In addition, the treatment of hepatitis C infections is currently advancing fast and therefore the costs are subjected to change. We determined the threshold value for each parameter at which it was no longer cost-effective or cost-saving. Lastly, we calculated at what price per condom and lubricant the intervention was still cost-saving, highly cost-effective and cost-effective.

### Sensitivity analyses

A sensitivity analysis was performed on the most important key parameters from Table 1. This was done by increasing and decreasing the parameters by 25%. This shows the effects of inaccuracies in the input data on the outcomes of the model. The results were depicted in a Tornado plot. As the outcomes plotted against total annual costs saved and the number of new HIV infections averted, the parameters on hepatitis C, chlamydia, gonorrhoea, and syphilis were excluded. The parameters ‘*Number of active MSM in the Netherlands*’, ‘*Percentage of active MSM, who met a casual partner at a PSV in the last year*’ and ‘*Prevalence among casual partners met in general HIV*’ had no effects on the outcomes and were therefore excluded as well. As the parameter ‘*Number of condoms distributed in 2013*’ was known exactly, it was not included in the model.

## Results

### Incidence of HIV, hepatitis C, chlamydia, gonorrhoea, and syphilis

We deduced that the annual incidence risks before intervention ranged from 0.0235% for hepatitis C to 27.4% for gonorrhoea (Table 2). Implementation of free condom distribution had largest effect on the less infectious viral infections; the annual incidence risk of HIV decreased with 7.62%, and that of hepatitis C with 7.14%. Whereas the decrease found among the more infectious bacterial infections was lower, the highest NIAs were found within this group (*n* = 394 for gonorrhoea and *n* = 261 for chlamydia).

**Table 2:** Annual incidences and number of cases of HIV, hepatitis C, chlamydia, gonorrhoea, and syphilis at public sex venues

	HIV	Hepatitis C	Chlamydia	Gonorrhoea	Syphilis
Annual incidence risk before intervention	3.43%	0.0235%	16.4%	27.4%	5.87%
Annual incidence risk after intervention	3.17%	0.0218%	15.4%	25.8%	5.49%
Decrease in annual incidence risk	7.62%	7.14%	6.32%	5.73%	6.46%
Incident cases before intervention	550	2.13	4128	6875	1473
Incident cases after intervention	508	1.98	3867	6481	1378
New infections averted	41.9	0.152	260.7	393.8	95.1

### Burden of disease

The estimated annual number of DALYs averted by the intervention was 207. The intervention had largest impact on HIV; 99% of DALYs averted were through prevention of HIV infections, while 0.14 and 0.64% were from preventing hepatitis C and the three bacterial infections combined (Table 3).

**Table 3:** Annual disability-adjusted life years lost due to HIV, hepatitis C, chlamydia, gonorrhoea, and syphilis at public sex venues

	DALYs lost before intervention		DALYs lost after intervention		DALYs averted	
	*n*	(%)	*n*	(%)	*n*	(%)
HIV	2691.7	(99.05)	2486.6	(99.04)	205.08	(99.22)
Hepatitis C	4.1	(0.15)	3.8	(0.15)	0.30	(0.14)
Chlamydia	6.1	(0.22)	5.7	(0.23)	0.39	(0.19)
Gonorrhoea	9.8	(0.36)	9.2	(0.37)	0.56	(0.27)
Syphilis	5.7	(0.21)	5.3	(0.21)	0.37	(0.18)
Total	2717.4		2510.7		206.69	

*DALY* disability adjusted life year

### Effects of increased condom use, condom effectiveness, and coverage

**Fig. 1:**
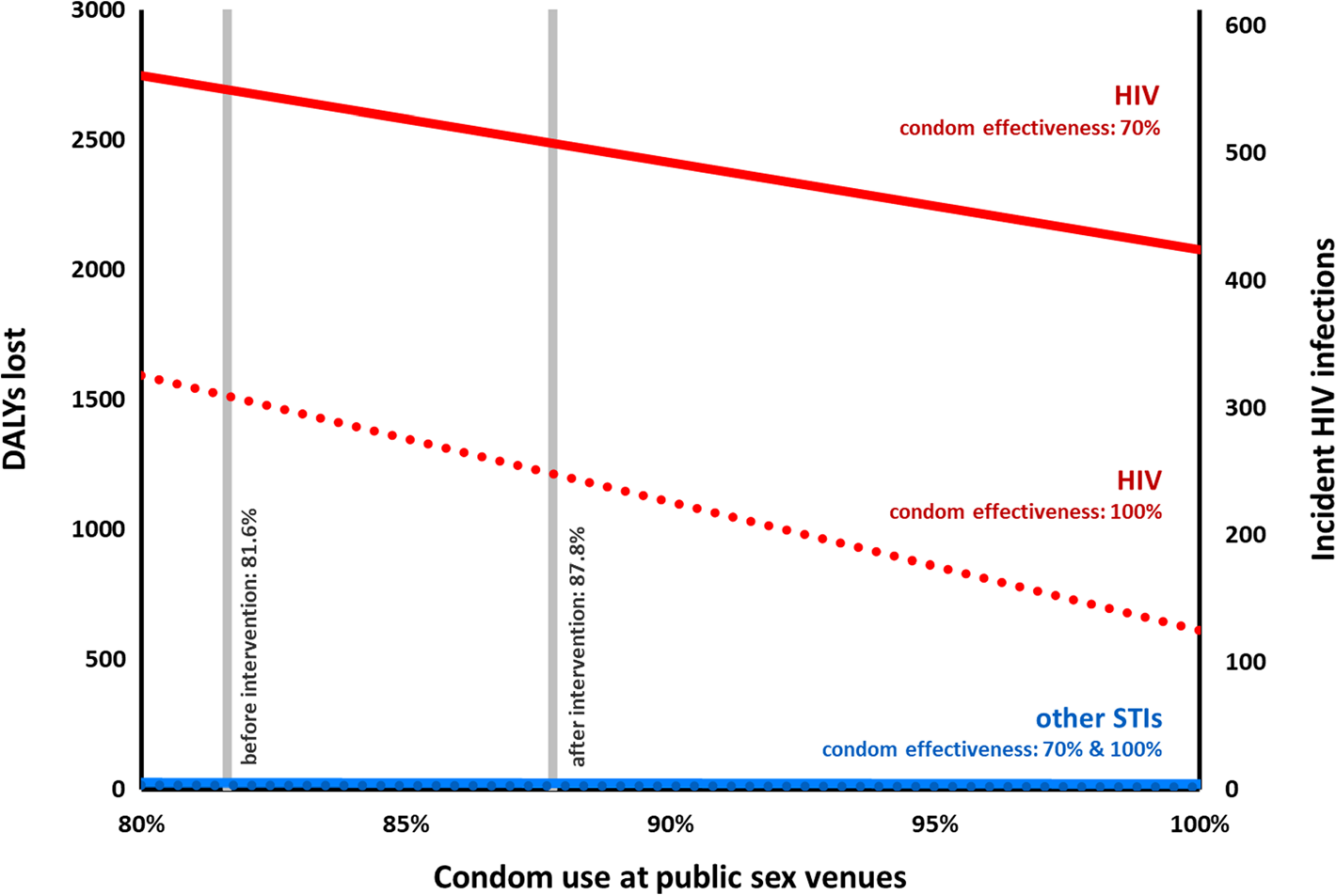
Graph of the relation between the number of disability-adjusted life years lost (left axis), the number of incident HIV infections (right axis) and condom use at public sex venues. Red lines indicate HIV, while blue lines indicate the cumulative effect on all other sexually transmitted infections (hepatitis C, chlamydia, gonorrhoea, and syphilis). Solid lines indicate the current condom effectiveness (70%), while dotted lines indicate a condom effectiveness of 100%. Condom use at PSV before and after the intervention are indicated by the vertical grey lines

**Fig. 2:**
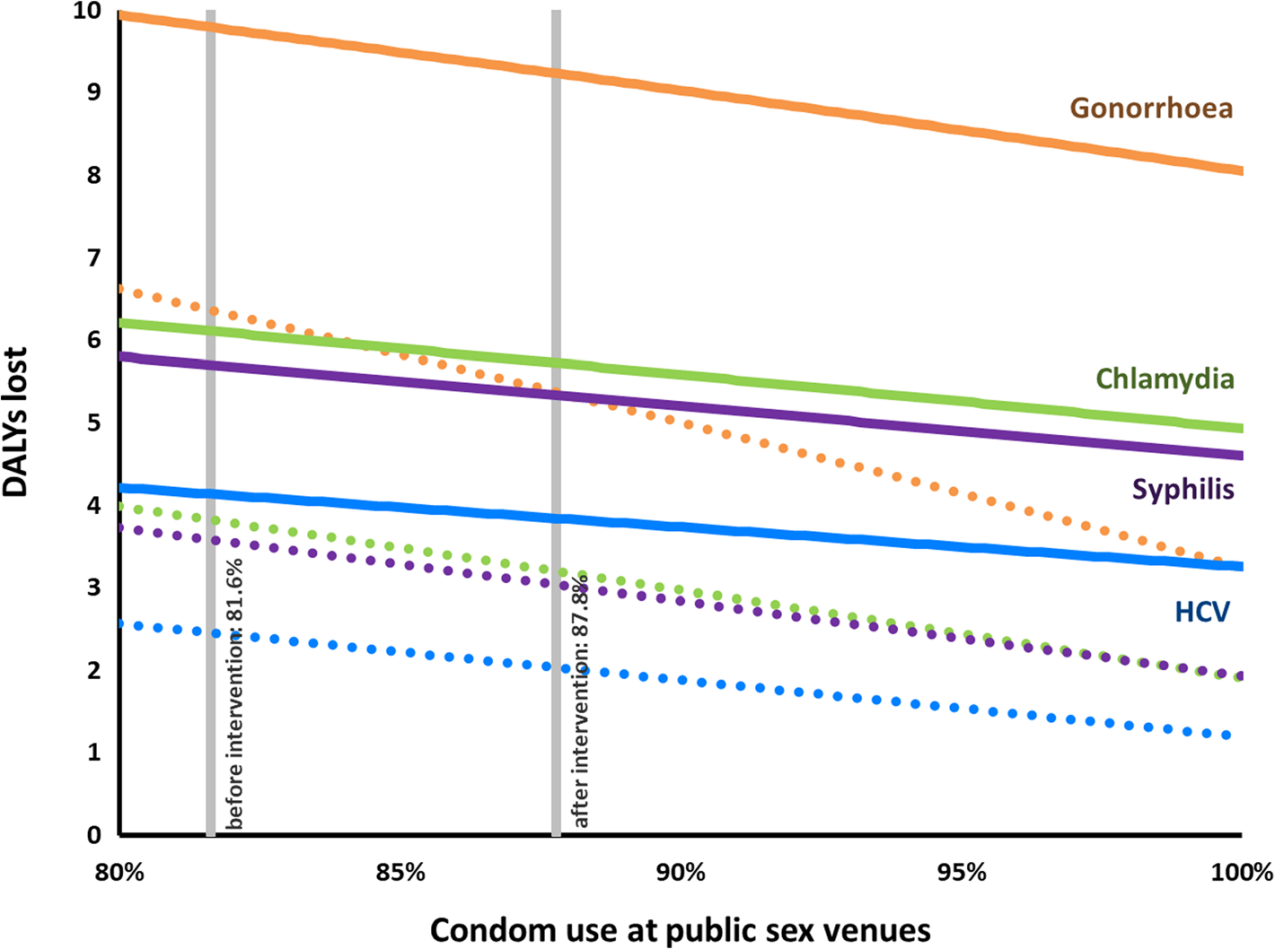
Graph of the relation between the number of disability-adjusted life years lost and condom use at public sex venues. The blue line indicates hepatitis C, the green line indicates chlamydia, the orange line indicates gonorrhoea, and the purple line indicates syphilis. Solid lines indicate the current condom effectiveness (70%), while dotted lines indicate a condom effectiveness of 100%. The condom use at PSV before and after the intervention are indicated by the vertical grey lines

Scenario studies were performed to examine the impact of increasing number of protected sexual acts at PSVs and increasing effectiveness of *CLub GUN* condoms. An increase in number of protected acts to 100% would result in a maximum of 126 HIV infections and 620 DALYs averted, while the number of condoms needed would increase with 13.9% to 423,743. If only the condom effectiveness was increased to 100%, 302 HIV infections and 1490 DALYs would be averted. If 100% of acts at PSVs were protected and condom effectiveness was 100%, the intervention would avert 425 HIV infections and 2098 DALYs. In this scenario, burden of disease of the five diseases combined would have decreased by 77.2% compared to the pre-intervention situation. The effects of upscaling condom use or effectiveness on disease burden of other STIs was minimal, compared to HIV, as the other STIs comprise between 0.94 and 1.34% of DALYs in all scenarios (Fig. 1). The largest decrease in DALYs can be seen for HIV, 77.3% at 100% condom use and 100% condom effectiveness. This decrease was a bit less for the other infections, ranging from 66.1% for syphilis to 71.1% for hepatitis C; Fig. 2).

Our models shows that 25,098 of the 51,415 eligible MSM in the Netherlands were included, which results in a coverage of 48.8%. An increase in coverage to 100% would add 104.9% to all NIAs and DALYs averted. In the scenario where both coverage and condom use were increased to 100%, the total number of condoms distributed increased with 133.4% to 868,071 condoms per year.

### Cost-effectiveness analyses and sensitivity analyses

In 2013, 371,952 condoms and 390,974 lubricants were distributed. As the total costs per distributed condom or sachet of lubricant were €0.07, the total costs of the *CLub GUN* project were €53,405. The maximum costs of the project, in the scenario where both coverage and condom use were increased to 100%, would be €124,638 annually, as 868,071 condoms and 912,465 lubricants would have to be distributed. As an estimated 207 DALYs were averted, costs per DALY averted was €258.38. The intervention was highly cost-effective, as gross domestic product (GDP) per capita (upper bound of costs per averted DALY for highly cost-effective interventions) was €35,864 [23]. It was estimated that 68% of HIV-infected MSM in the Netherlands were in care in 2013, of whom 85% are on cART [24]. Annual costs per HIV-infected MSM of consultations in an HIV treatment centre were €758, and €11,256 for those on cART [25]. Therefore the estimated average annual costs per HIV infection, including those undiagnosed, were €7026. The intervention averted 41.9 HIV infections, which would have cost €294,216 annually for consultations at HIV treatment centres and cART. This means that for every €1 spent, €5.51 was saved. Net costs saved would be €240,811: HIV treatment costs averted minus the costs of the intervention.

**Fig. 3:**
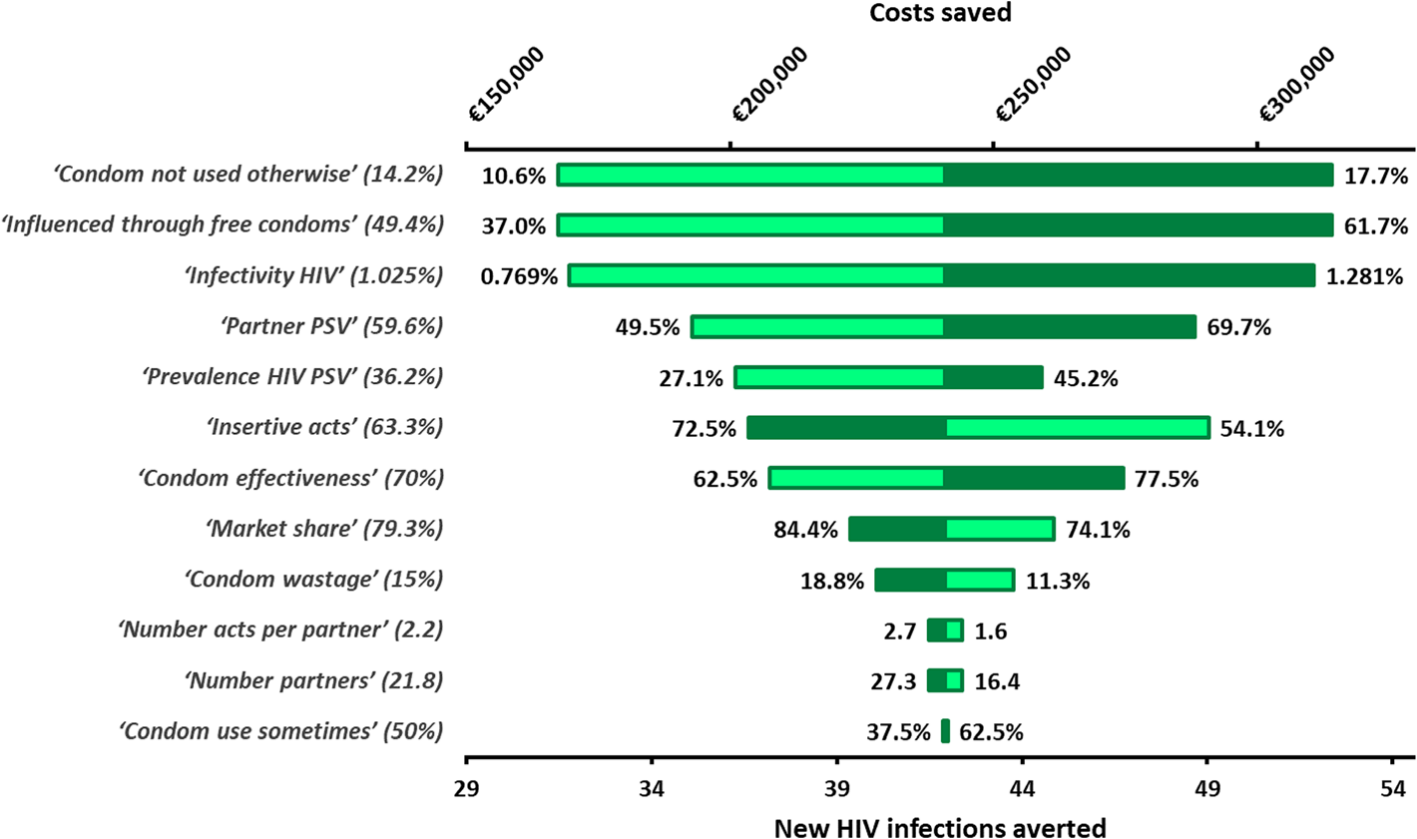
Tornado plot of the sensitivity analysis. All parameters were increased (dark green) or decreased (light green) with 25%. On the top axis the total annual costs saved can be seen, while on the bottom axis the annual number of new HIV infections averted is depicted. All parameters are described Table 1

A sensitivity analysis was performed for key parameters, which were increased and decreased by 25% (Table 1). In the sensitivity analysis, the intervention remained cost-saving for all parameter settings (Fig. 3).

For some parameters (*‘Condom use sometimes’*, *‘Insertive acts’*, and *‘Market share’*), the model remained cost-saving for every value between 0 and 100%, while for other parameters the model remained cost-saving up to extreme values (Additional file 4: Table S1). This effect was even greater, when analysing cost-effectiveness.

When increasing the total costs per condom or sachet of lubricant, the model remained cost-saving up to €0.39 per distributed item, 5.5 times the original costs. In addition, the model remained highly cost-effective or cost-effective up to €9.72 or €29.15 per item, 139 or 416 times the original costs respectively.

## Discussion

Distribution of free condoms at PSVs in the Netherlands is likely to have a substantial impact on the transmission of HIV. We estimated that the annual incidence risk of HIV among PSV patrons decreased with 7.62% due to the intervention, which corresponds to 42 new infections and 207 DALYs averted annually. Similar patterns were seen for the other infections, but due to the lower prevalence of hepatitis C and the lower severity of the bacterial infections, the reduction of these infections contributed only minimal to the reduction of burden of disease. This impact could potentially be even larger, as more beneficial results could be obtained by increasing condom uptake and especially condom effectiveness, as these factors have a large impact on modelled annual incidence risk of STIs. In addition, the current intervention is cost-saving, due to low costs of condoms and lubricants. For every €1 spent on condom distribution, €5.51 was saved, which leads to an estimated €240,811 that is saved annually on HIV-related medical expenses. This amount will increase even further when also the bacterial infections and hepatitis C are considered. Although no data is available on the actual costs saved, we are able to show the order of magnitude: in the Netherlands in 2011, 134 million euros of the national healthcare budget were spent on HIV and AIDS, while 56 million euros and 33 million euros were spent on STIs and viral hepatitis, respectively [26].

Other recent studies also showed the importance of condom use in HIV prevention: an increase in condom use showed an equal or greater effect on HIV transmission than an increase in HIV testing, linkage to care, and cART uptake combined [27, 28]. In agreement with our results, *Sadler* et al. found condom distribution projects can be cost-effective and cost-saving, especially in settings with high HIV prevalences such as MSM [29]. In these settings, they predicted that even a 2% increase in condom use would be cost-effective. This confirms that availability of free condoms at PSVs has a beneficial effect on transmission of HIV and other STIs, and on the financial burden this brings to society.

Although the current HIV prevention debate focuses much more on topics such as treatment as prevention and pre-exposure prophylaxis, condoms are the mainstay of STI prevention and should not be neglected. While pharmaceutical interventions will only effect HIV incidence, condoms work on all STIs, including those who still have to emerge. According to *Sullivan* et al., the most successful interventions combine efforts to increase condom use with other efforts such as increasing testing and treatment, as these interventions decrease STI prevalence and infectivity in the population [1]. Therefore, it is of great importance that the current *CLub GUN* project is integrated into HIV and STI prevention programs. Through collaboration with sexual health promotion institutes, information is available at PSVs on safe sex, testing, and treatment. Local public health services can provide general hygiene controls at PSVs, as well as easy access to testing facilities. For example, GGD Amsterdam is currently conducting STI testing at PSVs [30].

Most behavioural data in this study came from post-intervention measurements, as pre-intervention data were limited, and no clinical data on changes in annual incidence risk of the various infections during the intervention were collected. Therefore, the impact of the intervention could only be modelled and may deviate from the actual impact. Clinical data were derived from data collected at the STI outpatient clinic in Amsterdam. The specific clientele of the clinic may result in an overestimation of prevalence of HIV and other STIs. Visitors of STI outpatient clinics are expected to have a high risk for having an STI, even after correcting for having symptoms and partner notification. However, prevalences of bacterial STIs used in this study are similar to prevalences found at PSVs (chlamydia: prevalence in this study is 10.0% vs. 11.0% found in tested PSVs; gonorrhoea: 5.9% vs. 6.6%; syphilis: 1.8% vs. 1.1%) [16, 29], and number of HIV infections (*n* = 508) are in line with trends found among MSM in the Netherlands (700–750 newly identified cases annually) [31]. Another shortcoming of this study is the lack of data from more rural areas in the Netherlands. Most data, especially clinical and behavioural data, were gathered in Amsterdam and Rotterdam, and it is expected that the prevalence of STIs in more urbanised areas are higher than elsewhere in the Netherlands [32]. Most of participating PSVs are located in the larger cities, but a substantial part are spread throughout the Netherlands and their specific contribution to the transmission of HIV and other STIs remains unknown. Lastly, our static model is a simplified representation and might not contain all factors responsible for transmission dynamics of the various infections. No interactions between having an STI on susceptibility for HIV were considered, neither were risk reduction strategies like serosorting or seropositioning. These factors might be worth investigating in future models, as well as the use of a dynamic transmission model.

Nowadays HIV is considered a chronic infection instead of a lethal one, but the burden of HIV on MSM is still very high. The impact of STIs on the health of MSM is still dominated by HIV, and with an estimated prevalence of 36% among PSV patrons, these health concerns are still very real. Efforts to minimise the burden of disease due to HIV in MSM should therefore be continued unrelentingly. Our model shows that free condoms at PSVs could reduce the transmission of HIV substantially, as well as that of other STIs. A condom distribution program could be an affordable and an easily incorporated intervention to alleviate this burden. Future research should focus on how condom use and especially how condom effectiveness can be increased further, as these factors have a large impact on transmission of STIs. Most importantly, condom distribution projects should be made sustainable and incorporated into larger public health projects that focus on testing and treatment to maximise the effects of the intervention.

## Conclusions

Free condom distribution at public sex venues could reduce the transmission of HIV among men who have sex with men substantially. In addition, similar effects were seen for hepatitis C, chlamydia, gonorrhoea, and syphilis. Condom distribution could therefore be an affordable and easily implemented intervention that can reduce the burden of disease in men who have sex with men. However, this kind of intervention is likely to be most effective when combined with other efforts such as increased testing and treatment, as these interventions decrease the prevalence and infectivity of the various infections in the population. Future research should focus on how condom use and condom effectiveness can be increased further and how condom distribution projects can be incorporated into larger public health projects that focus on testing and treatment.

## Additional files

Additional file 1: **Data 1.** Calculations on condom use, number of MSM, condoms and casual partners, and prevalence and incidence. (PDF 242 kb)

Additional file 2: **Data 2.** Calculations on annual incidence risk. (PDF 118 kb)

Additional file 3: **Data 3.** Calculations on burden of disease. (PDF 284 kb)

Additional file 4: **Table S1.** Threshold values of the key parameters at which free condom distribution at PSVs remains cost-saving, highly cost-effective or cost-effective. (PDF 110 kb)

## Abbreviations

cART: combination antiretroviral therapy
*CLub GUN*: *Condoms & Lubricant Gay United Netherlands*
DALY: Disability-adjusted life year
GDP: Gross domestic product
MSM: Men who have sex with men
NIA: New infections averted
PSV: Public sex venue
STI: Sexually transmitted infection
UAI: Unprotected anal intercourse
WHO: World Health Organization
