# Supplementary material for: The effects of free condom distribution on HIV and other sexually transmitted infections in men who have sex with men
Source: BMC Infect Dis. 2019 Mar 4;19:222. doi: 10.1186/s12879-019-3839-0 (PMC6399837; doi:10.1186/s12879-019-3839-0)
Supplement: Supplementary file 1 — Data 1. Calculations on condom use, number of MSM, condoms and casual partners, and prevalence and incidence. (PDF 242 kb) [file 12879_2019_3839_MOESM1_ESM.pdf]

SUPPLEMENTARY DATA 1

The effects of free condom distribution on HIV and other sexually transmitted infections in men who have sex with men

Reinier J.M. Bom, Kalja van der Linden, Amy Matser, Nicolas Poulin, Maarten F. Schim van der Loeff, Bouko H.W. Bakker, and Theodoor F. van Boven

Condom use

I. Condom use at PSVs after intervention [1]:

Never :	**4.1%**
Sometimes :	**16.2%**
Always :	**79.7%**

Assumption: The MSM reporting "*Sometimes*" are assumed to use condoms 50% of the time. This is analysed in the sensitivity analyses as the parameter ***'Condom use sometimes'***.

II. Condom use in PSVs after intervention:

87.8%, 95%CI [83.8%, 91.0%]

based on **I.**
$= 79.7\% + 50\% \times 16.2\%$
$= f_{psvpost}$

III. Source of condom [1]:

Free condom, PSV (condom distribution) :	**39.0%**
Free condom, PSV (not condom distribution) :	**4.7%**
Free condom, elsewhere :	**9.7%**
Bought condom, PSV :	**29.3%**
Bought condom, elsewhere :	**5.8%**
Sex partner provided condom :	**11.3%**

Assumption: MSM reporting having used a free condom available at the PSV, are assumed to be influenced in their use through the availability of free condoms at the location. Those reporting bringing a free condom or buying a condom, are assumed not to be influenced. Condoms provided by the sex partner are excluded, as the source of these is unclear and assumed to be similar that of the index partner.

IV. Percentage of MSM that used a condom that were influenced through the availability of free condoms at PSVs:

49.4%, 95%CI [43.9%, 54.8%]

based on **III.**
$= (39.0\% + 4.7\%)/(100\% - 11.3\%)$
= ***'Influenced through free condoms'***

V. Percentage of MSM that used a free condom from the condom distribution, and that reported they would not have used a condom if condoms were not freely available at PSVs [1]:

14.2%, 95%CI [9.4%, 20.3%]

= ***'Condom not used otherwise'***

VI. Percentage of use unaffected by availability of free condoms:

93.0%

based on **IV.** & **V.**
$= 49.4\% \times (100\% - 14.2\%) + (100\% - 49.4\%)$

VII. Condom use in PSVs before intervention and in general:

81.6%

based on **II.** & **VI.**
$= 87.8\% \times 93.0\%$
$= f_{psvpre}$
$= f_{gen}$

VIII. Percentage of relationships with casual partners that involved anal intercourse formed at PSVs among men frequenting PSVs [2]:

59.6%, 95%CI [56.5%, 62.7%]

$= \delta$
= ***'Partner PSV'***

Number of MSM, condoms and casual partners

IX. Number of active MSM in the Netherlands [3]:

300,000, estimate

X. Percentage of active MSM, who met a casual partner at a PSV in the last year [4]:

17.1%, 95%CI [15.5%, 18.9%]

XI. Number of MSM eligible for inclusion in the intervention:

51,415

based on **IX.** & **X.**
$= 300{,}000 \times 17.1\%$

XII. Number of condoms distributed in 2013 [5]:

371,952, exact

XIII. Condom wastage:

15%, estimate

= ***'Condom wastage'***

XIV. Actual number of condoms used:

316,159

based on **XII.** & **XIII.**
$= 371{,}952 \times (100\% - 15\%)$

XV. Mean annual number of casual partners among men frequenting PSVs [4]:

21.8, [1–200]

= ***'Number partners'***

XVI. Mean number of sexual acts per casual partner [2]:

2.20, [1–50]

= ***'Number acts per partner'***

XVII. Mean annual number of sexual acts with casual partner:

48.0

based on **XV.** & **XVI.**

$= 21.8 \times 2.20$

$= n$

XVIII. Mean annual number of sexual acts at PSVs with casual partner:

28.6

based on **VIII.** & **XVII.**

$= 59.6\% \times 48.0$

XIX. Mean annual number of protected sexual acts at PSVs with casual partner:

25.1

based on **II.** & **XVIII.**

$= 87.8\% \times 28.6$

XX. Percentage insertive anal intercourse per sexual act [2]:

63.3%, 95%CI [59.2%, 67.2%]

= ***'Insertive acts'***

XXI. Market share of *CLub GUN* condoms at the *CLub GUN* locations:

79.3%, 95%CI [73.8%, 84.0%]

= ***'Market share'***

XXII. Mean annual number of *CLub GUN* condoms used per PSV patron:

12.6

based on **XIX.**, **XX.** & **XXI.**

$= 25.1 \times 63.3\% \times 79.3\%$

XXIII. Number of MSM included in the intervention:

25,098

based on **XIV.** & **XXII.**
= 316,159/12.6

XXIV. Coverage:

48.8%

based on **XI.** & **XXIII.**
= 25,098/51,415

Prevalence and incidence

XXV. Condom effectiveness [6,7]:

70%, estimate

$= \varepsilon$
= ***'Condom effectiveness'***

XXVI. Prevalence among casual partners met at a PSV [2]:

HIV : **36.2%**, 95%CI [29.9%, 42.8%]

$= p_{psvhiv}$
= ***'Prevalence HIV PSV'***

HCV : **0.46%**, 95%CI [0.05%, 2.11%]

$= p_{psvhcv}$

Chlamydia : **10.0%**, 95%CI [6.6%, 14.5%]

$= p_{psvchlam}$

Gonorrhoea : **5.9%**, 95%CI [3.4%, 9.7%]

$= p_{psvgono}$

Syphilis : **1.8%**, 95%CI [0.6%, 4.3%]

$= p_{psvsyph}$

XXVII. Prevalence among casual partners met in general [2]:

HIV :	**22.8%**,	95%CI [19.9%, 26.0%] $= p_{genhiv}$
HCV :	**0.40%**,	95%CI [0.11%, 1.08%] $= p_{genhcv}$
Chlamydia :	**9.6%**,	95%CI [7.6%, 11.8%] $= p_{genchlam}$
Gonorrhoea :	**6.1%**,	95%CI [4.5%, 8.0%] $= p_{gengono}$
Syphilis :	**2.0%**,	95%CI [1.2%, 3.2%] $= p_{gensyph}$

XXVIII. Per-act infectivity [8–12]:

HIV :	**1.025%**,	estimate $= \lambda_{hiv}$ = ***'Infectivity HIV'***
HCV :	**0.5%**,	estimate $= \lambda_{hcv}$
Chlamydia :	**17%**,	estimate $= \lambda_{chlam}$
Gonorrhoea :	**50%**,	estimate $= \lambda_{gono}$
Syphilis :	**30%**,	estimate $= \lambda_{syph}$

XXIX. Formula annual incidence risk:

based on **II.**, **VII.**, **XVII.**, **XXV.**, **XXVI.**, **XXVII.** & **XXVIII.**
see ***Supplementary Data 2***

$$\boldsymbol{I} = \Big(1 - (1 - (1 - \varepsilon_{psv})\, p_{psv}\lambda)^{n\delta f_{psv}} \times (1 - p_{psv}\lambda)^{n\delta(1-f_{psv})}\Big)\,\delta + \Big(1 - (1 - (1 - \varepsilon_{gen})\, p_{gen}\lambda)^{n(1-\delta) f_{gen}} \times (1 - p_{gen}\lambda)^{n(1-\delta)(1-f_{gen})}\Big)\,(1 - \delta)$$

Table 1. Parameters used in the annual incidence risk calculations.

Parameter	Description	Condition	Value
f_{psv}	Condom use at PSV	before intervention	81.6%
		after intervention, normal use	87.8%
		after intervention, 100% use	100%
f_{gen}	Condom use in general	all	81.6%
δ	Relationships formed at PSV	all	59.6%
n	Number of sexual acts	all	48.0
ε_{psv}	Condom effectiveness at PSV	normal effectiveness	70%
		100% effectiveness	100%
ε_{gen}	Condom effectiveness in general	all	70%
p_{psv}	Prevalence at PSV	HIV	36.2%
		HCV	0.46%
		Chlamydia	10.0%
		Gonorrhoea	5.9%
		Syphilis	1.8%
p_{gen}	Prevalence in general	HIV	22.8%
		HCV	0.40%
		Chlamydia	9.6%
		Gonorrhoea	6.1%
		Syphilis	2.0%
λ	Per-act infectivity	HIV	1.025%
		HCV	0.5%
		Chlamydia	17%
		Gonorrhoea	50%
		Syphilis	30%

References

[1] Osté JP, Bakker BHW, Cremer SW: *Gezonde keuzes makkelijk maken: Onderzoek naar gratis condoomverstrekking in sekslocaties.* Amsterdam: Schorer en GGD Amsterdam; 2008.

[2] Matser A, Heijman T, Geskus R, de Vries H, Kretzschmar M, Speksnijder A, Xiridou M, Fennema H, Schim van der Loeff M: **Perceived HIV Status is a Key Determinant of Unprotected Anal Intercourse Within Partnerships of Men Who Have Sex With Men in Amsterdam.** *AIDS Behav* 2014, **18**:2442-56.

[3] Schorer: *Factsheet: HIV en SOA bij MSM (juni 2011).* Amsterdam: Schorer; 2011.

[4] Goenee M, Picavet C: **Beschermingsgedrag van mannen die seks hebben met mannen.** In *Een wereld van verschil: seksuele gezondheid van LHBT's in Nederland 2013.* Edited by De Graaf H, Bakker BHW, Wijsen C. Delft: Eburon; 2014:85-104.

[5] Condomerie: *Jaarverslag 2013.* Amsterdam: Condomerie; 2013.

[6] Pickles M, Foss AM, Vickerman P, Deering K, Verma S, Demers E, Washington R, Ramesh BM, Moses S, Blanchard J, Lowndes CM, Alary M, Reza-Paul S, Boily MC: **Interim modelling analysis to validate reported increases in condom use and assess HIV infections averted among female sex workers and clients in southern India following a targeted HIV prevention programme.** *Sex Transm Infect* 2010, **86**:i33-43.

[7] Smith DK, Herbst JH, Zhang X, Rose CE: **Condom Effectiveness for HIV Prevention by Consistency of Use among Men Who Have Sex with Men (MSM) in the U.S.** *J Acquir Immune Defic Syndr* 2015, **68**:337-44.

[8] Jin F, Jansson J, Law M, Prestage GP, Zablotska I, Imrie JC, Kippax SC, Kaldor JM, Grulich AE, Wilson DP: **Per-contact probability of HIV transmission in homosexual men in Sydney in the era of HAART.** *AIDS* 2010, **24**:907-13.

[9] De Carli G, Puro V, Ippolito G: **Risk of hepatitis C virus transmission following percutaneous exposure in healthcare workers.** *Infection* 2003, **31**:22-7.

[10] Xiridou M, Vriend HJ, Lugner AK, Wallinga J, Fennema JS, Prins JM, Geerlings SE, Rijnders BJ, Prins M, de Vries HJ, Postma MJ, van Veen MG, Schim van der Loeff MF, van der Sande MA: **Modelling the impact of chlamydia screening on the transmission of HIV among men who have sex with men.** *BMC Infect Dis* 2013, **13**:436.

[11] Morin BR, Medina-Rios L, Camacho ET, Castillo-Chavez C: **Static behavioral effects on gonorrhea transmission dynamics in a MSM population.** *J Theor Biol* 2010, **267**:35-40.

[12] Holmes KK, Sparling PF, Stamm WE, Piot P, Wasserheit JN, Corey L, Cohen MS, Watts DH, (Eds): *Sexually transmitted diseases.* 4th edition. New York: McGraw-Hill; 2008.
