# Supplementary material for: The effects of free condom distribution on HIV and other sexually transmitted infections in men who have sex with men
Source: BMC Infect Dis. 2019 Mar 4;19:222. doi: 10.1186/s12879-019-3839-0 (PMC6399837; doi:10.1186/s12879-019-3839-0)
Supplement: Supplementary file 2 — Data 2. Calculations on annual incidence risk. (PDF 118 kb) [file 12879_2019_3839_MOESM2_ESM.pdf]

SUPPLEMENTARY DATA 2

The effects of free condom distribution on HIV and other sexually transmitted infections in men who have sex with men

Reinier J.M. Bom, Kalja van der Linden, Amy Matser, Nicolas Poulin, Maarten F. Schim van der Loeff, Bouko H.W. Bakker, and Theodoor F. van Boven

Annual incidence risk

The annual incidence risk I of the various STIs (i.e. HIV, HCV, chlamydia, gonorrhoea, and syphilis) before (*pre*) and after (*post*) the introduction of free condoms at PSVs is the probability to be infected during the year. Let I_y be the event of being infected during the year. Hence

$$\begin{aligned} I &= \mathbb{P}\left(I_y\right) \\ &= 1 - \mathbb{P}\left(\overline{I_y}\right) \end{aligned} \tag{1}$$

where, for any event I, $\overline{I}$ is the complement of I. Hence, $\mathbb{P}\left(\overline{I_y}\right)$ is the probability of not being infected during the year. Let n be the annual number of sexual acts with casual partners, and I_i, represents being infected during the ith sexual act. Using this notations we derive:

$$\begin{aligned} \mathbb{P}\left(\overline{I_y}\right) &= \mathbb{P}\left(\bigcup_{i=1}^{n} \overline{I_i}\right) \\ &= \prod_{i=1}^{n} \mathbb{P}\left(\overline{I_i}\right) \\ &= \prod_{i \in \{\text{PAI}\}} \mathbb{P}\left(\overline{I_i}\right) \prod_{i \in \{\text{UAI}\}} \mathbb{P}\left(\overline{I_i}\right) \\ &= \prod_{i \in \{\text{PAI}\}} \left(1 - \mathbb{P}\left(I_i\right)\right) \prod_{i \in \{\text{UAI}\}} \left(1 - \mathbb{P}\left(I_i\right)\right) \end{aligned} \tag{2}$$

We need to introduce some more notations. Let p be the prevalence of infection, ε the condom effectiveness, λ the per-act infectivity and f the proportion of protected sexual acts (PAI or protected anal intercourse). When unprotected (UAI or unprotected anal intercourse), infection occurs when the partner is infected and the act is infective. Hence, during an unprotected act,

$$\mathbb{P}\left(I_i\right) = p \times \lambda \tag{3}$$

During sexual contact with a condom, infection occurs when the condom is ineffective, the act is infectious and the partner is infected, so that for such an act:

$$\mathbb{P}\left(I_i\right) = \left(1 - \varepsilon\right) p\lambda \tag{4}$$

The number of protected acts per year is $n \times f$ and the number of unprotected acts is $n\left(1 - f\right)$. If we consider sexual acts to occur independently, using (3) and (4), (2) can be rewritten:

$$\mathbb{P}\left(\overline{I_y}\right) = \left(1 - \left(1 - \varepsilon\right) p\lambda\right)^{nf} \times \left(1 - p\lambda\right)^{n(1-f)} \tag{5}$$

Considering (1) and (5) we obtain:

$$I = 1 - \left(1 - \left(1 - \varepsilon\right) p\lambda\right)^{nf} \times \left(1 - p\lambda\right)^{n(1-f)} \tag{6}$$

Sexual acts can take place either at PSVs or not. Consider a ratio δ of sexual acts taking place at PSVs. Let D be the event that sexual contact occurs at PSVs. Conditioning by D, and using the law of total probability we obtain:

$$\begin{aligned} I &= \mathbb{P}\left(I_y\right) \\ &= \mathbb{P}\left(I_y|D\right)\mathbb{P}\left(D\right) + \mathbb{P}\left(I_y|\overline{D}\right)\mathbb{P}\left(\overline{D}\right) \end{aligned} \tag{7}$$

The definition of δ gives directly

$$\mathbb{P}\left(D\right) = \delta \text{ and } \mathbb{P}\left(\overline{D}\right) = 1 - \delta \tag{8}$$

If n_1 is the number of sexual acts at PSVs and n_2 the number of sexual acts outside PSVs, than

$$n_1 = n\delta \text{ and } n_2 = n\,(1-\delta) \tag{9}$$

Using formula (6), (8), and (9), we derive

$$\begin{aligned} I &= \left(1-(1-(1-\varepsilon)\,p_{psv}\lambda)^{n\delta f_{psv}} \times (1-p_{psv}\lambda)^{n\delta(1-f_{psv})}\right)\delta \\ &+ \left(1-(1-(1-\varepsilon)\,p_{gen}\lambda)^{n(1-\delta)f_{gen}} \times (1-p_{gen}\lambda)^{n(1-\delta)(1-f_{gen})}\right)(1-\delta) \end{aligned} \tag{10}$$

where p_{psv} is the prevalence of infection at PSVs, f_{psv} the proportion of protected sexual acts at PSVs, p_{gen} the prevalence of infection in general, and f_{gen} the proportion of protected sexual acts in general.
