# Supplementary material for: The effects of free condom distribution on HIV and other sexually transmitted infections in men who have sex with men
Source: BMC Infect Dis. 2019 Mar 4;19:222. doi: 10.1186/s12879-019-3839-0 (PMC6399837; doi:10.1186/s12879-019-3839-0)
Supplement: Supplementary file 3 — Data 3. Calculations on burden of disease. (PDF 284 kb) [file 12879_2019_3839_MOESM3_ESM.pdf]

SUPPLEMENTARY DATA 3

The effects of free condom distribution on HIV and other sexually transmitted infections in men who have sex with men

Reinier J.M. Bom, Kalja van der Linden, Amy Matser, Nicolas Poulin, Maarten F. Schim van der Loeff, Bouko H.W. Bakker, and Theodoor F. van Boven

Burden of disease

DALYs of the various STIs were calculated using uniform age weights, and without discounting, using the following formula [1,2]:

$$\begin{aligned} DALY &= YLD + YLL \\ &= IcTpDwLd + IcTpLl \end{aligned}$$

Where YLD is years lived with disability, YLL is years of life lost, Ic is the annual number of incident cases, Tp is the transition probability of entering a disease stage, Dw is the disability weight of the disease stage, Ld is the duration of the disease stage in years, and Ll are the years lost due to premature death caused by infection. The life expectancy of healthy males in the Netherlands was 79.5 years in 2011 [3]. Generalised courses of infection of HIV, hepatitis C, chlamydia, gonorrhoea, and syphilis in Dutch MSM in presence of treatment were constructed and included in the models. Parameter estimates of these generalised courses of infection were taken from $Cassini\ et\ al$ [4] and are shown in $Table$ 1.

For HIV, the course of infection start with a persistent HIV infection that can lead to permanent antiretroviral (ARV) treatment, or to early symptomatic AIDS, terminal phase AIDS, and premature death. We assumed that HIV was acquired at age 37.9 years [5]. The course of infection with hepatitis C was assumed as follows: the infection starts with an acute stage, followed by a chronic stage, which leads first to compensated liver cirrhosis, then to decompensated liver cirrhosis, hepatocellular carcinoma, and ultimately to premature death. Sexually transmitted hepatitis C is only found

among HIV-infected MSM and we assumed that the infection was acquired at age 42.6 years [6]. Acute and chronic infections could be cleared or progress towards compensated liver cirrhosis. No further stages of the disease (decompensated liver cirrhosis, hepatocellular carcinoma and premature death) will be entered as this would exceed the life expectancy of 79.5 years. For chlamydia and gonorrhoea, complications can occur after infection, but all cases recovered due to either spontaneous clearance or treatment. Syphilis' course of infection is composed of three stages (primary, secondary and tertiary syphilis) and recovery can occur in every stage and is seen for all cases. DALYs per STI were estimated as follows:

HIV:

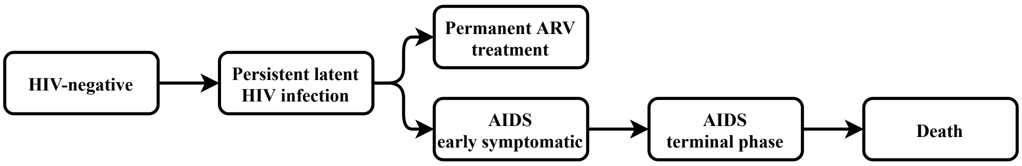

$$
\begin{aligned}
DALY_{hiv} &= Ic_{hiv}Dw_{phi}Ld_{phi} \\
&\quad +Ic_{hiv}Tp_{pat}Dw_{pat}Ld_{pat} \\
&\quad +Ic_{hiv}Tp_{aes}Dw_{aes}Ld_{aes} \\
&\quad +Ic_{hiv}Tp_{atp}Dw_{atp}Ld_{atp} \\
&\quad +Ic_{hiv}Tp_{d}Ll_{d}
\end{aligned}
$$

Hepatitis C:

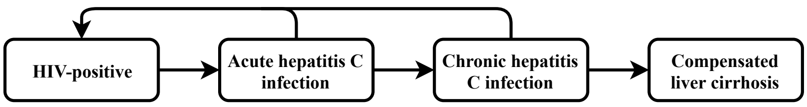

$$
\begin{aligned}
DALY_{hepc} &= Ic_{hepc}Dw_{ahci}Ld_{ahci} \\
&\quad +Ic_{hepc}Tp_{chci}Dw_{chci}Ld_{chci} \\
&\quad +Ic_{hepc}Tp_{cc}Dw_{cc}Ld_{cc}
\end{aligned}
$$

Chlamydia:

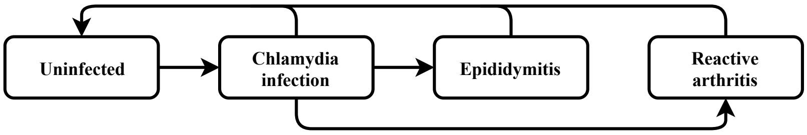

$$\begin{aligned} DALY_{chlam} &= Ic_{chlam}Dw_{ci}Ld_{ci} \\ &\quad + Ic_{chlam}Tp_{e}Dw_{e}Ld_{e} \\ &\quad + Ic_{chlam}Tp_{ra}Dw_{ra}Ld_{ra} \end{aligned}$$

Gonorrhoea:

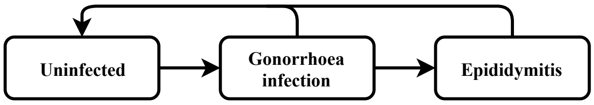

$$\begin{aligned} DALY_{gono} &= Ic_{gono}Dw_{gi}Ld_{gi} \\ &\quad + Ic_{gono}Tp_{e}Dw_{e}Ld_{e} \end{aligned}$$

Syphilis:

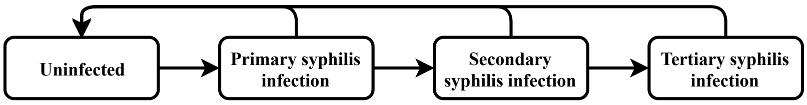

$$\begin{aligned} DALY_{syph} &= Ic_{syph}Dw_{psi}Ld_{psi} \\ &\quad + Ic_{syph}Tp_{ssi}Dw_{ssi}Ld_{ssi} \\ &\quad + Ic_{syph}Tp_{tsi}Dw_{tsi}Ld_{tsi} \end{aligned}$$

Table 1. Parameters used in the DALY calculations.

Infection	Disease stage	Transition probability		Disability weight		Duration	
**HIV**	Persistent HIV infection			Dw_{phi}	0.108	Ld_{phi}	9.27
	Permanent ARV treatment	Tp_{pat}	94.25%	Dw_{pat}	0.108	Ld_{pat}	32.33
	AIDS early symptomatic	Tp_{aes}	5.75%	Dw_{aes}	0.351	Ld_{aes}	5.36
	AIDS terminal phase	Tp_{atp}	1.85%	Dw_{atp}	0.574	Ld_{atp}	0.08
	Death	Tp_{d}	1.85%			Ll_{d}	26.89
**Hepatitis C**	Acute hepatitis C infection			Dw_{ahci}	0.013	Ld_{ahci}	0.13
	Chronic hepatitis C infection	Tp_{chci}	75.60%	Dw_{chci}	0.070	Ld_{chci}	36.50
	Compensated cirrhosis	Tp_{cc}	44.96%	Dw_{cc}	0.070	Ld_{cc}	0.27
**Chlamydia**	Chlamydia infection			Dw_{ci}	0.002	Ld_{ci}	0.02
	Epididymitis	Tp_{e}	15.56%	Dw_{e}	0.176	Ld_{e}	0.04
	Reactive arthritis	Tp_{ra}	0.31%	Dw_{ra}	0.402	Ld_{ra}	0.27
**Gonorrhoea**	Gonorrhoea infection			Dw_{gi}	0.051	Ld_{gi}	0.02
	Epididymitis	Tp_{e}	2.91%	Dw_{e}	0.176	Ld_{e}	0.08
**Syphilis**	Primary syphilis infection			Dw_{psi}	0.004	Ld_{psi}	0.31
	Secondary syphilis infection	Tp_{ssi}	23.00%	Dw_{ssi}	0.125	Ld_{ssi}	0.10
	Tertiary syphilis infection	Tp_{tsi}	0.30%	Dw_{tsi}	0.407	Ld_{tsi}	0.03

DALY = disability adjusted life year, ARV = antiretroviral.

References

[1] Murray CJ, Ezzati M, Flaxman AD, Lim S, Lozano R, Michaud C, Naghavi M, Salomon JA, Shibuya K, Vos T, Wikler D, Lopez AD: **GBD 2010: design, definitions, and metrics.** *Lancet* 2012, **380**:2063-6.

[2] Murray CJ, Vos T, Lozano R, Naghavi M, Flaxman AD, Michaud C, Ezzati M, Shibuya K, Salomon JA, Abdalla S, Aboyans V, Abraham J, Ackerman I, Aggarwal R, Ahn SY, Ali MK, Alvarado M, Anderson HR, Anderson LM, Andrews KG, Atkinson C, Baddour LM, Bahalim AN, Barker-Collo S, Barrero LH, Bartels DH, Basáñez MG, Baxter A, Bell ML, Benjamin EJ, et al.: **Disability-adjusted life years (DALYs) for 291 diseases and injuries in 21 regions, 1990-2010: a systematic analysis for the Global Burden of Disease Study 2010.** *Lancet* 2012, **380**:2197-223.

[3] World Health Organization: *World health statistics 2013.* Geneva: World Health Organization; 2013.

[4] Cassini A, Colzani E, Pini A, Mangen MJ, Plass D, McDonald SA, Maringhini G, van Lier A, Haagsma JA, Havelaar AH, Kramarz P, Kretzschmar ME, on Behalf of the BCoDE Consortium: **Impact of infectious diseases on population health using incidence-based disability-adjusted life years (DALYs): results from the Burden of Communicable Diseases in Europe study, European Union and European Economic Area countries, 2009 to 2013.** *Euro Surveill* 2018, **23**:16.

[5] Gras L, Geskus RB, Jurriaans S, Bakker M, van Sighem A, Bezemer D, Fraser C, Prins JM, Berkhout B, de Wolf F; ATHENA National Observational Cohort: **Has the rate of CD4 cell count decline before initiation of antiretroviral therapy changed over the course of the Dutch HIV epidemic among MSM?** *PLoS One* 2013, **8**:e64437.

[6] Vanhommerig JW, Lambers FAE, Schinkel J, Arends JE, Lauw FN, Brinkman K, Gras L, Rijnders BJA, van der Meer JTM, Prins M: **Risk Factors for Transmission of HCV Among HIV-Infected MSM: A Case-Control Study.** *Proceedings of the Conference on Retroviruses and Opportunistic Infections*; 2015 February 23-26; Seattle, US.
