# Supplementary material for: The effects of free condom distribution on HIV and other sexually transmitted infections in men who have sex with men
Source: BMC Infect Dis. 2019 Mar 4;19:222. doi: 10.1186/s12879-019-3839-0 (PMC6399837; doi:10.1186/s12879-019-3839-0)
Supplement: Supplementary file 4 — Table S1. Threshold values of the key parameters at which free condom distribution at PSVs remains cost-saving, highly cost-effective or cost-effective. (PDF 110 kb) [file 12879_2019_3839_MOESM4_ESM.pdf]

SUPPLEMENTARY TABLE 1

The effects of free condom distribution on HIV and other sexually transmitted infections in men who have sex with men

Reinier J.M. Bom, Kalja van der Linden, Amy Matser, Nicolas Poulin, Maarten F. Schim van der Loeff, Bouko H.W. Bakker, and Theodoor F. van Boven

Threshold values of the key parameters at which free condom distribution at PSVs remains cost-saving, highly cost-effective or cost-effective.

	Point estimate	Cost-saving	Highly cost-effective	Cost-effective
'Condom use sometimes'	50%	≥0%	≥0%	≥0%
'Influenced through free condoms'	49.4%	>8.9%	>0.35%	>0.12%
'Condom not used otherwise'	14.2%	>2.6%	>0.102%	>0.034%
'Partner PSV'	59.6%	>10.4%	>0.41%	>0.14%
'Condom wastage'	15%	<84.6%	<99.39%	<99.80%
'Number partners'	21.8	<885	<2624	<3503
'Number acts per partner'	2.2	<89	<264	<353
'Insertive acts'	63.3%	≤100%	≤100%	≤100%
'Market share'	79.3%	≥0%	≥0%	≥0%
'Condom effectiveness'	70%	>13.4%	>0.54%	>0.18%
'Prevalence HIV PSV'	36.2%	4.2%–95.3%	>0.019%	≥0%
'Infectivity HIV'	1.025%	>0.180%	≥0%	≥0%

PSV = public sex venue.
All parameters are described in detail in Supplementary data 1.
